# Non-Classical Congenital Adrenal Hyperplasia in Childhood

**DOI:** 10.4274/jcrpe.3378

**Published:** 2017-03-01

**Authors:** Selim Kurtoğlu, Nihal Hatipoğlu

**Affiliations:** 1 Erciyes University Faculty of Medicine, Department of Pediatric Endocrinology, Kayseri, Turkey

**Keywords:** Non-classical congenital adrenal hyperplasia, congenital adrenal hyperplasia, virilization, hirsutism

## Abstract

Congenital adrenal hyperplasia (CAH) is classified as classical CAH and non-classical CAH (NCCAH). In the classical type, the most severe form comprises both salt-wasting and simple virilizing forms. In the non-classical form, diagnosis can be more confusing because the patient may remain asymptomatic or the condition may be associated with signs of androgen excess in the postnatal period or in the later stages of life. This review paper will include information on clinical findings, symptoms, diagnostic approaches, and treatment modules of NCCAH.

## INTRODUCTION

Congenital adrenal hyperplasia (CAH) is a group of diseases which develop as a result of deficiency of enzymes or cofactor proteins required for cortisol biosynthesis (1,2,3,4,5). Due to cortisol deficiency, feedback control mechanism at hypothalamic and hypophyseal levels remains unsatisfactory, a defect which leads to an increase in adrenocorticotropic hormone (ACTH) production and consequently to adrenal hyperplasia (3). CAH is classified as the classical and non-classical types. The classical type constitutes the majority of the cases and results from 21-hydroxylase deficiency (21-OHD), which can present as the simple virilizing or as the salt-wasting types. While there is almost no enzyme activity in the cases presenting with salt wasting, the ratio of enzyme activity corresponds to 1-2% in simple virilizing types and to 20-50% in non-classical types (4).

Non-classical CAH (NCCAH) includes a series of diseases occurring due to gene mutations or disorders in the steroid synthesis steps of steroidogenic acute regulatory protein (StAR) providing the transfer of cholesterol from mitochondrial membrane to the cell. Although NCCAH occurs in the deficiencies of 21-OHD, 11β-hydroxysteroid dehydrogenase (11β-HSD) and 3-beta hydroxysteroid dehydrogenase (3β-HSD), and StAR mutations, it is most commonly observed in 21 and 11-b OHSD deficiencies. Its prevalence is reported as 1/1000 (6). However, the disease is observed in higher rates among Jewish, Mediterranean, Middle Eastern, and Indian societies (7).

Findings of genital virilization are not observed at birth in NCCAH patients. Although premature pubarche was detected in a 6-month-old infant as the earliest example, clinical findings and symptoms in NCCAH cases usually start from the age of 5 and usually emerge in late childhood, adolescence, and adulthood (8,9). Results of an analysis of 220 female patients with NCCAH showed that the clinical presentation started before the age of 10 in 11% and between the ages of 10-40 in 80% of the cases and that premature pubarche was the first symptom in 92% of the cases under 10 years of age (10). Increased androgen levels constitute the main basis of the clinical symptoms, but mild cortisol deficiency can also occur in some cases. Clinical findings include labial adhesion, perianal hair, clitoromegaly, increased penile length with prepubertal testicular volume, increased bone age, premature pubarche (development of pubic and axillary hair before the age of 8 and 9 for girls and boys, respectively), axillary apocrine odor, precocious puberty, greasy hair, acne, prepubertal gynecomastia, diffuse hair loss in centro-parietal and/or fronto-temporal regions, hoarseness of voice, menstrual irregularities (oligomenorrhea, anovulation, dysfunction), hirsutism, abortions, infertility in both genders, adrenal rest tumors, and short height according to midparental target height (11,12,13,14,15). In a prospective longitudinal study of Swedish children from birth to 18 years, it was demonstrated that permanent teeth eruption occurred at a mean age of 5.7+0.52 in these patients (16). Dental development may also occur at early ages (at ages 4-5 years) in these patients (17).

In a multicenter study including adolescents and adult females, hirsutism, oligomenorrhea, and acne were found in 59%, 54%, and 33% of the subjects, respectively (18). Tallness, accelerated bone maturation, development of premature pubic and axillary hair, and adult apocrine odor are findings which may be noted in both girls and boys in the prepubertal period. Heterozygote non-classical *21-hydroxylase* gene mutation was detected in 8.3% of the girls with premature pubarche (19). *CYP21A2* mutation was detected in 4.7% of the 126 subjects (122 girls, 4 boys) with premature pubarche, hirsutism, or polycystic ovary syndrome (PCOS) presentation (20). Increased penile length can be observed among boys. Prepubertal gynecomastia and adrenocortical incidentaloma are very rare findings detected in male cases (21,22). However, some NCCAH cases may be asymptomatic. In a study (23) which analyzed 330 family members of cases with mutation in terms of phenotype/genotype, a homozygote or compound heterozygote mutation was found in 51 relatives, and 42 of these relatives were clinically asymptomatic. A heterozygote mutation was found in 242 cases and it was observed that 37 cases were unaffected. In this study population, the most common genotypes for homozygote and compound heterozygote mutations were the V281L and V281L/IVS2-13A/C>G, respectively. The parents of the children whose diagnosis was certain were detected as undiagnosed symptomatic individuals.

Pubertal girls affected with NCCAH typically present with hirsutism (24,25). It is speculated that functional changes occur in the hypothalamic-pituitary-ovarian axis of these patients and that these changes lead to increased progesterone and/or 17-hydroxyprogesterone (17-OHP), androgens, 5-a reductase expression in the ovaries and/or directly lead to an increase in corticoid production. Excessive androgen impairs the progesterone sensitivity of the hypothalamus and increases luteinizing hormone (LH) secretion with rapid gonadotropin-releasing hormone (GnRH) pulses (26). Production of androgen from ovarian theca cells increases due to LH hypersecretion and contributes to the hyperandrogenemia. NCCAH due to 21-OHD was detected in 4.9% of 123 adult females who presented with severe heavy acne (27). Two studies on NCCAH from Turkey are worth mentioning. In one study, it was reported that among 285 females with a presentation of premenopausal hyperandrogenemia in Central Anatolia, the frequency of NCCAH due to 21-OHD was 2.1% (28). In another study performed in a similar area, 9.52% of 63 hirsutism cases (43 and 20 of these cases were diagnosed as PCOS and idiopathic hirsutism, respectively) were found to have non-classical 21-OHD (29). Akinci et al (30) have reported that among adolescent patients (age range 13 to 19 years) with hirsutism, 21-OHD NCCAH was detected in only one case (3%). It is known that in utero androgen exposure occurs in classical CAH cases, but this is not observed in NCCAH cases (21).

### Diagnostic Studies in Non-classical Congenital Adrenal Hyperplasia

It may be difficult to distinguish the clinical symptoms and findings of premature adrenarche from those of PCOS in girls (20). Although a high 17-OHP level is diagnostic in classical CAH cases, this finding may be insufficient for a diagnosis of NCCAH. Therefore, the ACTH test is accepted as the gold standard for a diagnosis of NCCAH.

### Non-classical 21-OHD:

It should be noted that basal 17-OHP should be measured in the morning hours (06:00-08:00 a.m.) on an empty stomach and at the follicular phase in menstruating females (between the 3^rd^ and 5^th^ postmenstruation days) (21). This is because the 17-OHP value exceeds 2 ng/mL in the luteal phase in half of healthy females (31). Boys suspected of NCCAH should be tested immediately (32). A basal value between 1.7 and 3.0 ng/mL is sufficient for diagnosis (31,33,34). In a study in which late-onset 21-OHD was detected in a rate of 3.2% in 186 children diagnosed with premature pubarche, a basal 17-OHP level of 1.55 ng/mL was suggested as the cut-off value (35). The consensus regarding basal 17-OHP concentration for ACTH test indication is reported as 2 ng/mL. A basal 17-OHP level over 5 ng/mL is regarded as a quite high value (14). NCCAH was found in 4.2% of 238 French children with premature adrenarche and it was understood that a basal 17-OHP level over 2 ng/mL demonstrates 100% sensitivity and 99% specificity (36). After estimation of basal 17-OHP levels, ACTH is applied intramuscularly or intravenously in a dose of 250 mg/1.73 m^2^ and a second sample is taken after 60 minutes. The majority of researchers agree that a 17-OHP level over 10 ng/mL at the 60^th^ minute of ACTH application is a criterion for diagnosis of late-onset 21-OH deficiency and this conclusion is in agreement with results of genetic studies (37). However, some authors suggest that 12 ng/mL should be the cut-off limit (5). A 21-deoxycortisol value in addition to 60^th^ minute 17-OHP level with ACTH test for late-onset 21-OHD being exceeding 400 pg/mL is also taken as criterion (38). ACTH and corticotropin-releasing hormone (CRH) are not high in these cases. Furthermore, total and free testosterone levels and the levels of sex hormone-binding globulin (SHBG), cortisol, and 11-deoxycortisol should be measured at baseline and at the 60^th^ minute. Generally, the value of 60^th^ minute cortisol with ACTH test is expected to be >18 mg/dL. However, it is also important to detect the cases below this limit. Stoupa et al (39) reported that cortisol values measured in the ACTH test were below 18 mg/dL in 60% of 47 children with late-onset 21-OHD. If the cortisol level remains below 18 mg/dL, it should be noted that these cases may be under risk of adrenal deficiency in stress situations.

### Non-classical 11b-HSD deficiency:

Basal 11-deoxycortisol level is over 10 ng/mL in late-onset 11-OH deficiency (40). According to Reisch et al (40), cut-off values for basal deoxycortisol level are 6.95 ng/mL and 7.23 ng/mL for prepubertal and pubertal cases, respectively. When 60^th^ minute 11-deoxycortisol level is higher than 18 ng/mL in the ACTH test, the diagnosis becomes definitive (5).

### Non-classical 3b-HSD deficiency:

In 3b-HSD cases, the criteria for diagnosis consist of a basal 17-OH pregnenolone level above 30 ng/mL and a 17-OH pregnenolone/cortisol ratio above 10 SD (5). In girls with oligomenorrhea, anti-Müllerian hormone (AMH) increase may be detected before hyperandrogenemia (41).

Another point to take into account during basal tests is presence of a secondary biosynthetic defect. Eldar-Geva et al (42) detected 3b-HSD, 21-OH, and 11b hydroxylase mutations in 12.3%, 10%, and 8% of 170 females presenting with hirsutism, respectively. They observed partial 11b-OH deficiency in 21-OH cases and partial 3b-HSD deficiency in 11b-HSD cases. These authors suggested that this incident which they named as secondary biosynthetic defect may be associated with intra-adrenal accumulating androgens.

Another point to discuss about diagnostic criteria is the measurement methods. Around the world in general, 17-OHP and 11-deoxycortisol levels are usually measured by immunoassay [radioimmunoassay (RIA), immunochemiluminometric assay (ICMA), electrochemiluminescence immunoassay (ECLIA)] methods. Interference is a common problem in RIA methodology, so there is a need for improvement in purification and extraction methodology. Recently, more reliable and accurate results were reported with liquid chromatography coupled with mass spectrometry (LC-MS/MS) devices (41,43). Ambroziak et al (44) reported a study in which hormone levels were measured by immunoassay and LC-MS/MS methods in 39 females with hyperandrogenism presentation and 29 females in a control group. Total testosterone, dehydroepiandrosterone sulfate (DHEA-S), androstenedione, and 17-OHP were measured with immunoassay and LC-MS/MS methods and it was understood that the values measured with immunoassay methods were higher. It was reported that 85% of the patients were subjected to unnecessary tests and investigations due to high 17-OHP. Moreover, analyzing the urinary steroid metabolites of the cases with capillary gas chromatography/mass spectrometry in selective ion monitoring mode (GC/MS-SIM) device is important. The diagnosis can be definitive by measuring a 17-OHP metabolites in the urine, namely 17-hydroxy pregnenolone (17-OHP, normal value is 63-279 mg/24 hours), pregnanetriol (PT, normal value is 179-992 mg/24 hours), 21-deoxycortisol metabolite pregnanetriolone (PTN, normal value is 3.5-50 mg /24 hours) (44). In addition to these tests, these same authors conducted urinary steroid metabolite studies and genetic tests on 40 adult female patients (age range of 18-39 years) with hyperandrogenism presentation. An ACTH test was conducted in patients with a basal 17-OHP level of 1.7-10 ng/mL. The cases detected to have a basal and post-ACTH stimulation 17-OHP value over 10 ng/mL were subjected to genetic testing for urinary metabolites in 24-hour urine and late-onset 21-OH deficiency type. 17-OHP levels of 21 cases were determined to be over 10 ng/mL after ACTH, but only five of the cases (24%) were diagnosed definitely with NCCAH by using urinary steroid profile and genetic data. The diagnoses based on basal and post-ACTH 17-OHP levels over 10 ng/mL at the end of the study are not conclusive, and since 75% of the results are false-positive, it is suggested that definitive diagnosis should be obtained by using urinary steroid profile and genetic studies (45). Another point to be noted is that 11-deoxycortisol and cortisol demonstrate a cross-reactivity with a rate of 23.3% among measurement methods. Thus, Xu et al (46) reported that the level of Immulite 2000 and cortisol can be false-low, whereas 11-deoxycortisol level can be false-high.

### Genetic Studies

Genetic studies in classical and non-classical CAH cases are needed. Siblings and parents should also subjected to genetic analyses. Since the most common type is 21-OH, *CYP21A2–CYP21A1P* mutations are generally investigated. Null 12 G splice, 1172N, P3OL, V28IL, P453S.Int2 mutations are observed most commonly and they are positive in 73-87% of the cases (14,22,34,47). NCCAH phenotype is detected in 98 % of the cases with mutation V28IL. 11-b hydroxylase (*CYP11b1*) and 3-b hydroxysteroid dehydrogenase (*HSD3b2*) and StAR mutations observed more rarely should be studied (48).

### Differential Diagnosis

Tumors producing androgen leading to premature pubarche presentation, androgen exposure, premature adrenarche, cortisone reductase deficiency, and DHEA sulphosphotransferase deficiency should be considered in the differential diagnosis (9,21). Indeed, a case diagnosed with NCCAH in early years was reported to have an ovarian steroid cell tumor (49).

The cases observed to have StAR mutation (lipoid adrenal hyperplasia) known as NCCAH can be considered to be cases of familial glucocorticoid deficiency (50). Also, if enzyme P450SCC separating the side chain of cholesterol undergoes a partial defect, it can be mistaken for lipoid adrenal hyperplasia which is a kind of NCCAH (51).

### Screening

It is known that NCCAH cases usually cannot be detected during CAH screenings made in the neonatal period. Held et al (52) have reported that detection rates of non-classical 21-OHD at the first and second screenings during neonatal screening for CAH were 1/217 573 and 1/32 465, respectively.

### Treatment Planning

If bone age is found to be advanced in a prepubertal girl or boy with NCCAH, final height loss can be prevented by stopping the progress with hydrocortisone. At this decision, diagnosis, bone age, and time of start of treatment need to be considered. Cases in whom treatment was initiated one year prior to onset of puberty and who had a bone age below 9, final height remained within the genetic potential (53). The generally accepted approach is to initiate hydrocortisone treatment in cases observed to have prepubertal growth acceleration or apparent advancement of bone age. A second indication for starting treatment with hydrocortisone depends on the finding that the cortisol level measured at the 60^th^ minute of the ACTH test does not exceed 18 mg/dL (54).

Another issue is to administer growth hormone (GH) and GnRH agonist treatment for their additive effect on final height. In a study where GH and GH+GnRH agonists were given to prepubertal 3 cases and pubertal 3 cases, improvement in predictive heights were observed when compared to an untreated group (55). However, it is generally accepted that routine GH and GnRH should not be given to these patients before the emergence of the signs and symptoms of central precocious puberty or unless predictive adult height is below 2 SD value of the average population value (54).

Hydrocortisone is administered in a dose of 6-15 mg/m^2^/day, divided into 3 doses (21). In treatment of adolescent patients with hydrocortisone, several points need to be considered. First, in adolescents, compliance tends to decrease with time. Secondly, while the half-life of hydrocortisone corresponds to 80 minutes during pre/post-pubertal period, it falls to 40 minutes in puberty (2). This is because the increasing IGF-1 level decreases 11-b OHSD type-1 activity and also increases cortisol clearance due to increase in glomerular filtration rate. Adrenal would be suppressed with the administration of hydrocortisone, so in cases of inflammatory disease, surgical operation, and trauma, corticoid should be administered in stress doses (21). When the patients receiving hydrocortisone treatment reach adolescence, the treatment can be ended if there are no findings of hyperandrogenism such as hirsutism, acne, and oligomenorrhea. At this time, an ACTH test is also done to control hypothalamic-pituitary-adrenal axis. If hyperandrogenism findings are clear, administering 0.25 mg of dexamethasone at night is recommended (54). Some clinicians suggest that hydrocortisone treatment should be continued for another 2-3 years in the post-menarcheal period, since girls initially have anovulatory cycle after menarche (2). When boys reach Tanner stage 3 (testis volume 8-10 mL), hydrocortisone is discontinued and normal development of pubertal height is ensured (2). If peak cortisol level of pubertal and adult females measured after ACTH is below 18 mg/dL, steroid treatment is administered only in cases of stress. Insufficiency of adrenomedullary functions does not occur in these cases (56). However, if an increase in levothyroxine or hyperthyroidism occurs, adrenal crisis may arise as the result of increased clearance of cortisol (57). In cases with hirsutism, treatment with oral contraceptive agents leads to an increase in SHBG production in the liver, to a decrease in androgen release from the ovary, and consequently to improvement in menstrual irregularity (14). If necessary, anti-androgens (spironolactone, flutamide, cyproterone acetate, or finasteride) may be added to the treatment. Cosmetic approaches such as laser application and depilatories can also be suggested (2,15,21). In some cases, hydrocortisone treatment can be continued if oral contraceptives and anti-androgens cannot be tolerated or when hyperandrogenemia is quite severe (2).

### Transfer to Adult Endocrine Units

The issue of transferring the patients who have completed their adolescent years to adult endocrine units is usually neglected. In this transfer process, the medical and social problems of each patient needs to be well investigated and the information needs to be transferred in detail. The “Kieler Modell” created by Kruse et al (58) can be taken as an example on this subject. The process of transferring to adulthood takes place at ages 17-18 years. It is suggested that contact informing meetings attended by the pediatric and adult teams should be held a year before this process and that the cases should be monitored together. Moreover, the specialists of endocrinology, gynecology, urology, and psychiatry should be encouraged to participate in the meetings organized to introduce the cases and share the information (54,58).

### Long-term Problems

In NCCAH cases, a series of problems such as acne, oligomenorrhea, hirsutism, abortion-stillbirth, deep voice in women, infertility, impaired life quality, psychiatric problems (psychosis, suicide, alcohol use, drug use), decrease in bone density, fractures, obesity, dyslipidemia, insulin resistance, diabetes, increase in the thickness of intima media, hypertension, cardiovascular problems, early mortality risk, and tumorigenesis risk may arise (14,59). Although testicular adrenal rest tumors are observed in classical CAH cases, it is reported that they may also occur in NCCAH cases (60). It is known that rest tissue originates from adrenal stem cells. In some cases, when the treatment is insufficient, testicular adrenal rest tumors develop with ACTH stimulation. These tumors are hypoechoic and smooth marginated. Infertility occurs as a result of testosterone production deficiency and oligospermia as a result of mass compression. Therefore, periodic testis ultrasonograhic follow-up is suggested for NCCAH cases (60). Development of rest tissue and tumor in ovaries is less frequent (61). NCCAH may also be the cause of adrenal incidentaloma cases, but the frequency of this complication is not known (15). Androgenic or feminizing adrenal tumors can rarely be seen in NCCAH cases (62,63).

### Fetal Problems

The overall rate of miscarriages in NCCAH patients is 20%. NCCAH also increases the risk of classical and non-classical CAH for the offspring babies of affected mothers (18). For these reasons, having more information about this genetics-based condition is very important, particularly in communities where kin marriage is frequent.
